# Sixth-Year Molars

**Published:** 1895-11

**Authors:** N. Pearson

**Affiliations:** Toronto, Ont.


ARTICLE IV.

### SIXTH-YEAR MOLARS.

BY N. PEARSON, L. D. S., TORONTO, ONT.

Read before Toronto Dental Society.

Can this tooth be saved? If you say it can be saved, then I say do so. If you say it cannot be saved, then I say save it as long as you can. Save the remnants as long as the filling will be of any use, and after this put on a crown and save the roots.

This tooth is presented to us under varying circumstances. Let us examine some of them. First of all a child is brought to us after an attack of toothache. The mother (as is usual) has been kept awake all night, perhaps several; the child can't eat, has had no sleep, nor no one else. This is the usual round of explanation, and the end of it is—as an instruction—to take out the tooth and have done with it. The child is between six and twelve years of age. Now what argument is there advanced or to be conjectured why this tooth should be removed? One only, and that is, to relieve present pain, which is no argument at all, but simply an experiment, and should never be entertained either in this or any other case. To remove any tooth because it aches, simply is to forget that we are a profession and decline to the days of turnkeys and forceps, and the years when they constituted all that there was in it. I say it is no excuse to extract a tooth because it pains. Well, have we any arguments as to why the tooth should not be extracted? Yes. First, because it is not necessary to do so, as the desired result can be brought about in other ways. Second, because this tooth occupies a position pre-eminently destined by nature to fulfill a great work at a critical period in the growth and development of the child—a period in which the child is not amenable to reason. It would be no use to say to the child, "Now that you have lost this tooth, you will have to compensate by a careful use of the other side and see that your digestion does not suffer from its loss." I apprehend thet the child would open its eyes, and we could read in big letters some such word as "Rats!" or "What are you giving us!" printed on its astonished face. This tooth was destined to take on hard work at the earliest period of its appearance, both by relieving the temporary molars which may soon begin to be defective, and because the constitution is beginning to demand more solid food and greater variety of diet It is the connecting link between the milk teeth and the perfect mill of mastication soon to be appropriated. These teeth are the advanced guard, wisely prepared by a great General to do duty in an important field. They are the pioneers which are intended to occupy the ground and erect the bulwark of the physical frame and constitution of the child, and act as defenders of and contributors to its general development. Are we the enemies then that are to fly in the face of that All-wise Providence and mutilate His work, destroy His designs, and frustrate His plans? Rather, I should say, we are His creatures intended to help and further His plans, to assist and encourage by every means in our power the natural laws which govern existence and work out to perfection, so far as we can, the means provided to this end. This is mere sentiment and will go just so far with scientific men. Well, aside from this, let us consider the human side. Did it ever occur to any one of you that the taking out of this tooth is a violent, inhuman and most barbarous proceeding; all this because it is not necessary, not intended by nature, and not provided for as is the case with the deciduous teeth. I now ask the question, Ought these teeth to be saved? Should the attempt be made to save them? All I have said is in the affirmative, speaking from the point of use. I now add to the argument the further very large argument in the make-up of the features. The central position of these teeth and their induction at this time of life has a very marked influence upon the features. The first teeth are never irregular. Nature provides for the wants of the milk teeth, yields to their demands for sufficient accommodation and provides the space. How does she do it? By adapting the teeth to the size of the circle, or by adapting the circle to the size and number of the incoming teeth? The jaws are enlarged to suit the demands of the teeth.

Ordinarily the natural inception of the sixth-year molar will be met by a growth of bone, a prolongation of the circle to meat the demand, and, by the time of its full development, the jaw and the alveolus have been added to the original formation to just the extent required for this tooth, all tending to add to maturity, expression and facial development and symmetry. Here we have situated the largest tooth in the formation, central as regards mastication, first to get to work in grinding, almost invariably perfect antagonism, never irregular, and, in fact, the keystone of the whole arch, put there designedly, intended for work, and come to stay. Unfortunately it is not always perfect; sometimes very imperfect, often troublesome, generally weak, subject to infirmities so are children, always the object of solicitation, and a stumbling-block to the professional, amateur, and "others." I now add emphatically to the *saving* argument, that this tooth should never be removed for regulating purposes, except in rare and very exceptional cases, and never before the appearance of the twelfth-year molar. I am not speaking by the book, I am now speaking from close observation for many years particularly directed to this point. For years I directed my course by printed instructions from standard authority. I extracted sixth year molars where there was reason to doubt a prolonged existence or a slight deflection in the anterior teeth. I don't do it any more. I never looked at the roots of a first molar without a sigh of regret, a tinge of shame, and a professional blush, and I don't do it any more—any more than I can't avoid. I bend my energies to avoiding it, and generally succeed. In the early days of my hallucination for extracting these teeth I made victims of my three eldest children, and can never forgive myself for it. I present you for inspection several models which speak for themselves; also, by contrast, you may compare other models before you with these teeth *in situ.* I need not comment on either.

These teeth are removed inconsiderately and indiscriminately—without consideration as to the effect upon the facial expression or use in mastication, and without discrimination as to proportion or malposition of other teeth.

It is frequently the victim of malice propense, some of the best authorities and most competent writers holding that where a predisposition to caries exists, it is advisable to anticipate its loss by early extracting, and prepare the way for its successors to climb into its position, and in the near future, or at the age of twenty, say, the teefh are in better state than would be by a doubtful first molar. If a competent dentist sits down and examines the four first molars as to all the points involved, and concludes that there is no use in trying to save them, the parent is then absolved from responsibility; but I should like to know upon what grounds he is acting before I make up my mind that there is no other way out of it. The same prolongation and development of bone takes place at the eruption of the second and third molars, speaking in a general way. A gradual development is brought about from the time of the demand for more room by the incoming first molar until the appearance of the last one, when it ceases. We remove the first molar any time between the seventh and twelfth years, and further development ceases until the space is occupied and further demand is made by the wants of the third molar for accommodation in the circle. It is a very difficult matter to determine just how much has been added in each case, or just how much the natural development has been interfered with by premature extraction, but it does seem to me that there must be considerable deviation from normal state in view of the fact that a slight change of position of any one or more teeth, such as is done sometimes in regulating, alters facial expression, and want of symmetry is dependent upon such slight changes as we are able to produce by a little pressure judiciously applied.

I apprehend that no difference of opinion would exist in regard to these teeth in a general way where they are well-developed, reasonably good and easily treated, and due attention exercised upon them by parents and the dentist. It is when, by ignorance and negligence, they are allowed to become objects of care and annoyance that our attention is directed to them.

Now, supposing that we are convinced in our own minds that they "ought to be saved," the next point is, can they be saved? I think they can, generally, not invariably. A good deal depends upon the operator. If the dentist says "that the tooth is better out," the responsibility is taken off the parents' hands and remains with the dentist. Whether right or wrong, wise or unwise, he shoulders all the consequences. Whether he is doing this to hide his own weakness or inability, or a conscientious conviction of the advisability of the course, the consequences are on his head. If he says that the tooth may be saved and made useful, the reasonable conclusion is that he has a reason for the hope that there is in him and the onus of the situation and condition that the tooth is in lies with the parties responsible previous to his cognizance. The dentist does his best to save the tooth, and at all events will no doubt succeed in relieving present pain, which is the beginning of the end.

I am aware of a case in Toronto which was taken to a father in dentistry, whose professional lexicon commences with the word *extract* in large capitals, followed by such other words as extermination, extinction and substitution. In the particular case I am referring to he was persuaded, against his will, to endeavor to save the tooth. After such treatment as he thought advisable, he put in an amalgam filling, with the instruction that he might see the case if any further trouble ensued. The next step was to drill a hole below the gum margin. That tooth has been in active service for sixteen years, and lately yielded to rational treatment with a fair promise of beholding its great grandchildren in the dim future. Here is a model of teeth taken from a subject operated on by the same bright shining light, whose specialty is the "regulation of children's teeth."

Let me end this paper by a few conclusions which may or may not be orthodox. The designer of the turnkey was a great benefactor to the human race. Many no doubt there were who heaped benedictions on his inventive genius. Their day is passed. We shall never know the history of first molars during the five or six thousand years of pre-historic existence or what the custom was in the way of dealing with them, The inventor of forceps introduced a new era in dentistry; and many called him blessed.

It has been left to the days of the present generation and the introduction of high art in saving teeth during the last few years to minimize this agony, and we hope that the time may come when the ignorance of the parents will no longer be an excuse for their employment, and that by a combination of opportunity and intelligent application of the resources first molars may be allowed to fulfil their destiny.

One of the questions propounded to the class graduating in 1869 was, "What would you do with an abscessed tooth." The answer offered by a clever, well-educated Normal School graduate, "Jerk the thing out," apparently pleased the examiner, for he said he agreed with the senti ment. Better counsels prevail. Now-a-days we are on a higher plane, and such proceeding is no more necessary than removing a great toe for an ingrowing nail.

I am aware of some of the difficulties met with in every-day office practice. I sympathize with the perplexities of the dentist and the terror of the patient, also I feel for the unfortunate parent who, perhaps, is most to blame for the present state of things. I can't point to a road that should be universally travelled, except to say, that it is always a safe line to relieve the distress and fill temporarily, with the urgent understanding that you must be consulted frequently, and held responsible for consequences as to effects outside of the tooth proper. I can see no reason for early action in removing, except to allow early occupation of the field by incoming posteriors, which is not going to increase the masticating surface, but merely shift its locality. I do see where harm may be the result of injudicious removal to the features, to the masticating power, and to the general health and physical make-up of the patient.—*Dominion Dental Journal.*

